# Long-Term Venovenous Connection for Extracorporeal Carbon Dioxide Removal (ECCO_2_R)–Numerical Investigation of the Connection to the Common Iliac Veins

**DOI:** 10.1007/s13239-020-00466-y

**Published:** 2020-05-13

**Authors:** N. B. Steuer, K. Hugenroth, T. Beck, J. Spillner, R. Kopp, S. Reinartz, T. Schmitz-Rode, U. Steinseifer, G. Wagner, J. Arens

**Affiliations:** 1 Department of Cardiovascular Engineering, Institute of Applied Medical Engineering, Medical Faculty, RWTH Aachen University, Aachen, Germany; 2 Clinic for Cardiothoracic Surgery, University Hospital RWTH Aachen, Aachen, Germany; 3 Department of Anesthesiology, University Hospital RWTH Aachen, Aachen, Germany; 4 Department of Radiology, University Hospital RWTH Aachen, Aachen, Germany; 5 Institute of Applied Medical Engineering, Medical Faculty, RWTH Aachen University, Aachen, Germany; 6 Chair in Engineering Organ Support Technologies, Department of Biomechanical Engineering, Faculty of Engineering Technologies, University of Twente, Enschede, The Netherlands

**Keywords:** Cannulation, Anastomosis, Computational fluid dynamics (CFD), Extracorporeal life support (ECLS), Extracorporeal carbon dioxide removal (ECCO_2_R), Artificial lung, Recirculation

## Abstract

**Purpose:**

Currently used cannulae for extracorporeal carbon dioxide removal (ECCO_2_R) are associated with complications such as thrombosis and distal limb ischemia, especially for long-term use. We hypothesize that the risk of these complications is reducible by attaching hemodynamically optimized grafts to the patient’s vessels. In this study, as a first step towards a long-term stable ECCO_2_R connection, we investigated the feasibility of a venovenous connection to the common iliac veins. To ensure its applicability, the drainage of reinfused blood (recirculation) and high wall shear stress (WSS) must be avoided.

**Methods:**

A reference model was selected for computational fluid dynamics, on the basis of the analysis of imaging data. Initially, a sensitivity analysis regarding recirculation was conducted using as variables: blood flow, the distance of drainage and return to the iliocaval junction, as well as the diameter and position of the grafts. Subsequently, the connection was optimized regarding recirculation and the WSS was evaluated. We validated the simulations in a silicone model traversed by dyed fluid.

**Results:**

The simulations were in good agreement with the validation measurements (mean deviation 1.64%). The recirculation ranged from 32.1 to 0%. The maximum WSS did not exceed 5.57 Pa. The position and diameter of the return graft show the highest influence on recirculation. A correlation was ascertained between recirculation and WSS. Overall, an inflow jet directed at a vessel wall entails not only high WSS, but also a flow separation and thereby an increased recirculation. Therefore, return grafts aligned to the vena cava are crucial.

**Conclusion:**

In conclusion, a connection without recirculation could be feasible and therefore provides a promising option for a long-term ECCO_2_R connection.

## Introduction

Since its first successful application in 1971,^19^ extracorporeal life support (ECLS, at that time called extracorporeal membrane oxygenation (ECMO)^12^) has become an established treatment for circulatory and pulmonary support, and a combination thereof.^34^ ECLS was originally a modified cardiopulmonary bypass, the use of which was limited to short-term support only. The longer duration of support with ECLS was facilitated by the simplification of the circuit. Nonetheless, the development was mainly caused by the introduction of polymethylpentene (PMP) hollow fiber membranes.^14^

Yet, patients were still sedated and intubated. In the last 10–15 years, the application of ECLS has evolved to a mid-term support with awake and spontaneously breathing patients treated in the ICU.^4^ Again, this development was supported by technological advances, specifically in the field of oxygenators and pumps,^32^ and by novel double-lumen cannulae, making ECLS simpler and safer.^4^ As a result, the use of ECLS increased by 433% in the US from 2006 to 2011.^43^

Today, ECLS patients can be awake and ambulatory^4^ and treated for prolonged time periods up to several months or occasionally even longer.^3^,^29^,^36^,^45^,^50^ This enables the use of ECLS for new indications, for example as bridge to lung transplantation.^7^,^20^,^48^ Furthermore, new extracorporeal applications have emerged, such as extracorporeal carbon dioxide removal (ECCO_2_R) In general, the ambulation of patients is associated with better outcomes at hospital discharge,^44^ which has also been confirmed for ECLS.^1^,^30^

In the future, as postulated by Bartlett and Deatrick,^4^ ECLS will be wearable thus facilitating a management in step-down units, general care or even at home and increasing the quality of life for patients. Additionally, ECLS will be used as destination therapy, at first for the palliation of severe COPD. Yet, for the realization of this next milestone in ECLS therapy, further technological improvements are needed. Current research focuses on the development of wearable and integrated devices, such as the MLung,^47^ PAL^33^ and RasQ.^8^

However, these new integrated and wearable devices designed for ambulatory long-term treatment are in need of improved connections to the patient’s vessels.^23^ Currently used cannulae are associated with several complications and disadvantages, which prolong applications. These include cannula thrombosis,^6^,^9^,^24^ limb ischemia,^13^,^21^,^22^,^27^,^38^,^42^,^51^ risk of dislocation,^21^,^38^ infection of the cannulation site,^13^,^22^,^38^ limitation of patient’s mobility,^13^,^21^,^22^,^38^ high effort of ambulation,^13^,^21^,^22^,^49^ and arguably a low quality of life.

Therefore, we propose a new venovenous (VV) connection to the patient’s common iliac vessels (CIV), which is implemented using grafts. The iliac anastomosis offers the possibility of an extraperitoneal approach in analogy to a kidney transplant. We hypothesize that this connection could provide sufficient blood flow for elective interventions for long-term lung support of chronic patients, such as ECCO_2_R, without disturbing the physiological blood flow. The grafts could exit in the abdominal region without hindering the patient’s mobility. When connecting to the iliac vessels, it is possible to guide the grafts to the exit site without puncturing the peritoneum. The connection is realized as an end-to-side anastomosis, a technique that has been proven to be long-term stable for connections of ventricular assist devices.

In this study, we investigate the connection of grafts to the CIVs for venovenous ECCO_2_R. Both drainage and return grafts are connected to a CIV on either side before the convergence to the inferior vena cava (IVC). Since both grafts are connected to the same blood vessel and in proximity of each other, the blood returned from the extracorporeal circulation (ECC) to the patient may be directly withdrawn back to the ECC by the drainage graft Since both grafts are connected to the same circulation and in proximity of each other, the returned blood may be directly withdrawn by the drainage graft. This process is called “recirculation”^22^,^42^ and is suspected to be a major issue of the proposed connection.

Recirculated blood through the ECC entails a decreased gas exchange efficiency, which is detrimental—especially in small devices for ambulatory applications. Therefore, it was selected as the primary focus of the study. Wall shear stress (WSS) was selected as the secondary focus of the study, as it allows conclusions to be drawn about the severity of the iatrogenic changes to the blood flow. Both factors were evaluated to deduce the possibility of a connection configuration that avoids recirculation and induces only minimal changes to the physiological blood flow.

In summary, we hypothesize that it is possible to connect the drainage and return of an ECC to the left and right CIV, respectively, using grafts, without recirculation and maintaining a physiological blood flow.

## Materials and Methods

The establishment of an anatomical model was needed for both the numerical simulation of the flow characteristics of the novel ECCO_2_R connection and the subsequent *in*-*vitro* validation thereof.

### Anatomical Model

**Figure 1:**
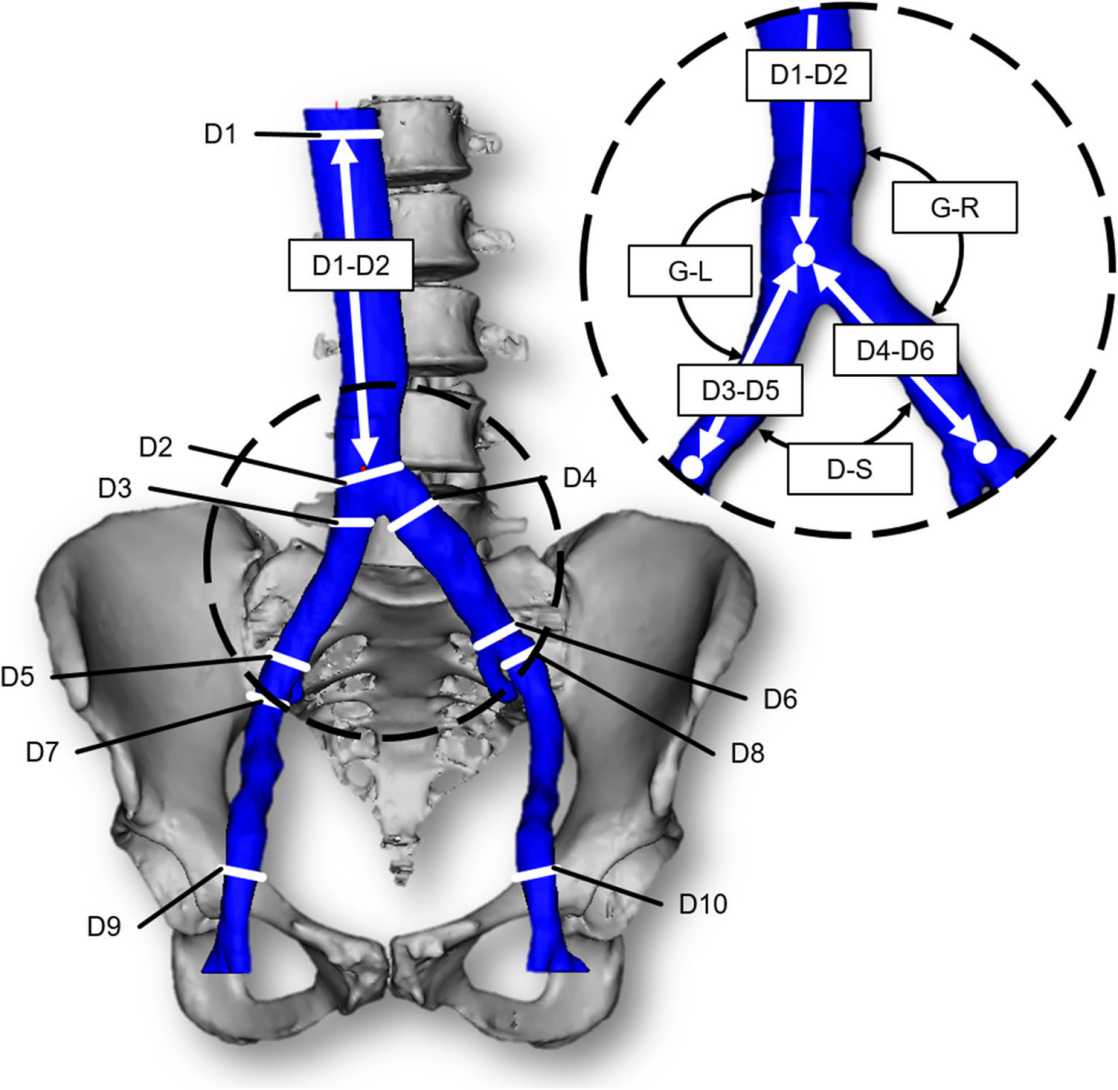
Measurement locations with corresponding denominations in one exemplary anatomy.

To identify a suitable anatomical model for the site of connection, imaging data of patients in supine position were analyzed and evaluated. A board-certified radiologist selected CT data of 24 patients with healthy vascular systems. All imaging data were anonymized. Three data sets were excluded due to insufficient image quality, two data sets due to abnormal anatomy of the venous vessels, and one data set due to an extreme deformation of the spine. We segmented and measured the remaining 18 data sets at distinctive locations (Fig. 1). Therein, D1 is the diameter of the IVC, measured at the level of the first lumbar vertebra and D2, also the diameter of the IVC, but measured just after the convergence of the CIVs to the IVC. D3, D4, D5, and D6 are diameters of the CIVs: D3 (right branch) and D4 (left) are measured just before the convergence into the IVC whereas D5 (right) and D6 (left) are measured just after the convergence of the external and internal iliac vein into the CIVs. D7, D8, D9, and D10 are diameters of the external iliac vein, whereby D7 (right) and D8 (left) are measured just before the convergence of the external and internal iliac vein into the CIVs and D9 (right) and D10 (left) are measured just after the transition from the femoral vein to the external iliac vein. Additionally, we measured the distances between D1 and D2, D3 and D5 (length of right CIV), and D4 and D6 (length of left CIV). Furthermore, the angles between IVC and each CIV as well as the angle between both CIVs were measured. The segmentation and measurement of the diameters and distances were performed using Mimics Research 20.0 (Materialise NV, Leuven, Belgium). For the measurement of the angles, we exported the centerline into Creo Parametric 4.0 (PTC Inc., Boston, MA).

The arithmetic mean, median, standard deviation, median absolute deviation, and coefficient of variation were calculated for each measurement of a diameter, distance, and angle. To identify a suitable patient anatomy for the evaluation of the novel connection, the percentage deviation from the average value for each data set for each measurement was calculated. Thereafter, the average percentage deviation was calculated for each data set and the one with the lowest value was chosen as the most suitable anatomy. This anatomy was transferred into 3-matic Research 13.0 (Materialise NV, Leuven, Belgium), where it was smoothed and fixed to eliminate e.g. holes and overlaps.

## Computational Fluid Dynamics

The chosen anatomical model was supplemented by two vascular grafts (DesignModeler, Ansys 19.0, ANSYS, Inc., Canonsburg, PA) in order to evaluate different connection configurations by numerical simulation: the drainage graft on the left CIV and the return graft on the right CIV. Both grafts were connected with an angle of 25°, as this was previously found to be beneficial and to entail near-physiological WSS.^17^,^18^ We implemented the grafts as ideally straight tubes and assumed a perfect anastomosis with an oval orifice for all simulations. Three parameters were varied in the numerical simulations:

- Diameter of the grafts
- Distance of the grafts to the iliocaval junction (IJ)
- Position of the grafts, defined by the rotational angle around the native flow direction

The diameter of the grafts was varied between 7 and 9.5 mm. For the distance of the grafts to the IJ, we chose two discrete positions. One position was located close to the IJ, denoted as “high” and one was located more caudally, denoted as “low”. Both the drainage and return positions were varied independently, which yielded four different setups. We were unable to implement a program controlled variation of the distance because we used a physiological anatomy, which is complex, skewed, and tortuous. Therefore, the location of the intersection of graft and CIV would vary, which led to the termination of the simulations either because of a faulty, or a failed connection of the CIV and the graft. In each of the four setups, the rotational angles were restricted either by the opposing CIV or graft and therefore, the limits had to be established individually.

**Figure 2:**
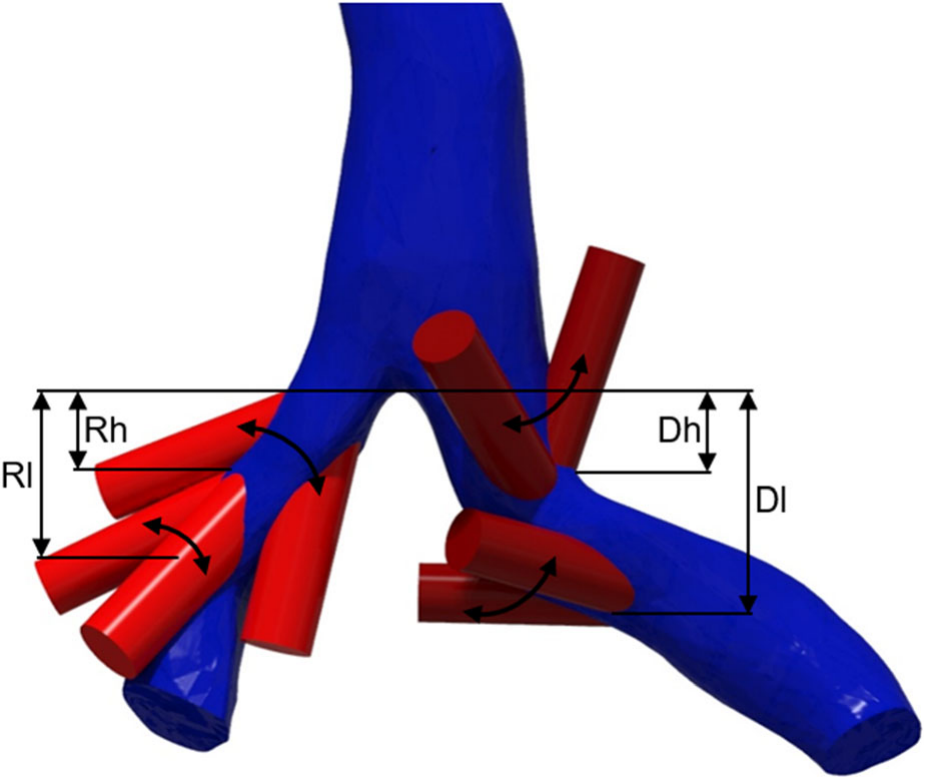
Schematic presentation of the rotational angles of the grafts and the respective distances to the IJ.

The different setups and the corresponding distances and ranges of the rotational angles are shown in Table 1. Figure 2 provides a schematic depiction of the rotational angles of the grafts and the respective distances to the IJ.

**Table 1:** The different setups for the CFD simulations including the distance of the grafts to the IJ and the range of the rotational angle.

Setup	Distance to IJ (mm)		Range of rotational angle (°)	
	Return	Drainage	Return	Drainage
Return high, Drainage high (Rh_Dh)	27	26	− 160–160	− 60–220
Return high, Drainage low (Rh_Dl)	27	39	− 160–160	− 10–245
Return low, Drainage high (Rl_Dh)	38	26	− 100–100	− 60–210
Return low, Drainage low (Rl_Dl)	37	39	− 110–100	0–240

The distance to the IJ was measured from lowest point of the IJ to the furthest intersection point of CIV and graft.

The first part of the numerical study was designed to investigate the influence of the different parameters. Therefore, 20 design points were created for each of the chosen setups using Optimal-Space-Filling-Design (Ansys 19.0, ANSYS, Inc., Canonsburg, PA). This function facilitates the generation of sensibly distributed configurations for optimal parameter correlation. Subsequently, CFD simulations were performed for all created design points. Based on these simulations, a parameter response surface was created from which the influence of the different parameters on the recirculation can be derived. The second part of the numerical study was designed to determine the maximum and minimum values for the recirculation. For this purpose, we used the optimization function provided by Ansys and chose the screening method with 1,000 samples for each setup. The response surface is used to calculate additional candidate points without simulation and thus identifies graft configuration with minimum and maximum recirculation. The identified optimal candidate points and results were validated by additional CFD simulations and added to the overall design points for the evaluation. Additionally, the WSS on the vessel wall for each design point was calculated.

For this study, the return blood flow in the CIVs was set to 1.0 L/min each, as the lower margin of the physiological range. For the drainage blood flow at the IVC, a pressure boundary condition with 6.0 mmHg was chosen. We investigated two different scenarios, represented by different blood flows through the grafts: On the one hand, a critical but still acceptable scenario, in which the flow through the grafts is at the upper end of blood flows clinically used in ECCO_2_R (0.5–1.0 L/min).^16^ This resulted in a graft flow of 100% of the CIV flow. On the other hand, we chose a worst-case scenario, in which the patient’s demand for extracorporeal CO_2_ removal increases either due to a deteriorating lung function or an increased demand caused by the ambulation. Consequently, the blood flow through the ECC is increased, which can entail a graft flow that exceeds the native blood flow. This resulted in a graft flow of 150% of the CIV flow. Additionally, we assumed that this amplifies the recirculation as the corresponding CIV does not provide enough blood for the drainage and thereby more returned blood is drawn to the drainage graft. Therefore, the influences of the varied parameters on the recirculation are augmented, providing more insight into the effects. To allow a reasonable comparison to the validation experiments, blood was implemented as a Newtonian fluid with a dynamic viscosity of 3.6 mPas and a density of 1056.4 kg/m^3^, which corresponds to a hematocrit of 44%. For the investigation of recirculation two fluids with identical properties, as described above, were created. One fluid enters through both CIVs and the other one enters at the return graft. Thus, the recirculation is calculated as the ratio of the mass flow of the second fluid to the total mass flow through the drainage graft. All numerical simulations were performed steady state using Ansys CFX 19.0 (ANSYS, Inc., Canonsburg, PA). The RNG-κ-ε-Model was used to model turbulence.

**Figure 3:**
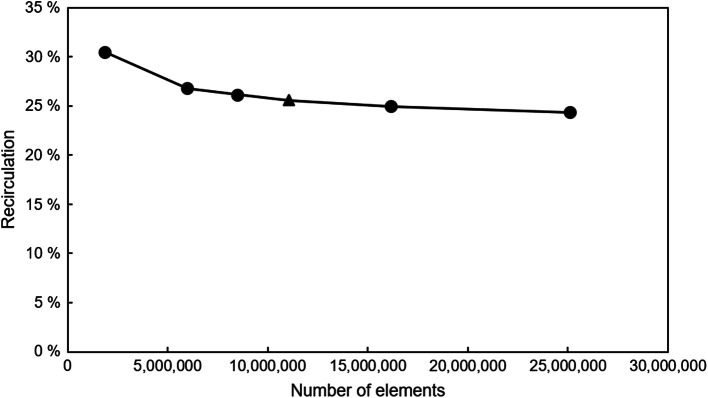
Recirculation dependent on the number of the elements of the 6 different meshes, used in the sensitivity study. The triangle represents the mesh, which we chose for the further analyses.

The geometry was meshed using Ansys Meshing 19.0 (ANSYS, Inc., Canonsburg, PA). The mesh parameters were set to: 5 inflation layers at the vessel and graft wall, growth rate of 1.2, and first layer thickness of 0.1 mm, to achieve a dimensionless distance from the wall of below 2 (y^+^ < 2). The global element sizing (0.3–0.8 mm) was varied to study the mesh sensitivity. This method was taken from Thamsen *et al*.^46^ Six meshes with different numbers of elements were compared regarding the results of the recirculation. Since this study focused on the investigation of a broad range of configurations, and with regard to a feasible computational time, a 5% deviation of the recirculation to the finest mesh was deemed acceptable in accordance with the literature (acceptance values 3–9%^2^,^15^). Thus, mesh 4 was chosen for the further analyses and additionally, an experimental validation was performed to ensure the validity of the results. The characteristics of the meshes, the resulting recirculations, and the percentage deviation of each recirculation to the result of the finest mesh are shown in Table 2. Figure 3 depicts the recirculation dependent on the number of elements of the meshes included in the sensitivity study.

**Table 2:** Characteristics, resulting recirculations, and percentage deviation of each recirculation to the result of the finest mesh of the different meshes used for the mesh sensitivity study.

Mesh	Global element size (mm)	Elements	Recirculation (%)	Percentage deviation (%)
1	0.8	1,848,658	30.47	20.08
2	0.5	5,978,652	26.80	9.15
3	0.44	8,484,479	26.12	6.76
4	0.4	11,065,390	25.52	4.60
5	0.35	16,132,503	24.94	2.36
6	0.3	25,090,090	24.35	0.00

### Validation Experiments

**Figure 4:**
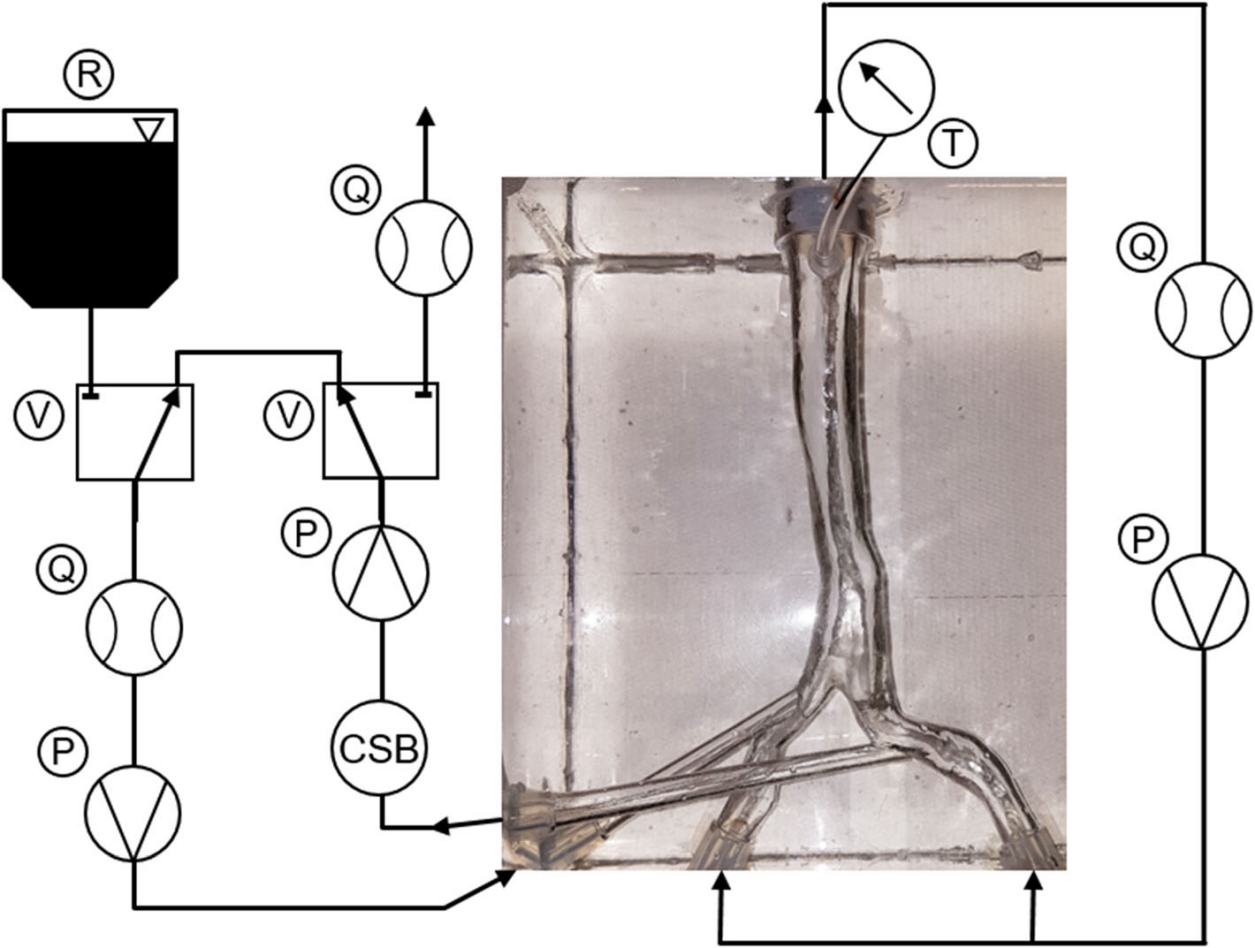
Experimental setup for the validation of the CFD simulations.

With one setup and both numerically investigated graft flows we performed dye experiments to validate our CFD simulations. We chose the setup Rh_Dl with a favorable position of the grafts for manufacturing and a high recirculation. The geometry was manufactured by rapid prototyping (Objet 350 Connex3, Stratasys, Ltd., Eden Prairie, MN) of a liquid acrylic-based photopolymer (VeroClear™, Stratasys). The support material was removed mechanically first roughly by hand and then chemically in a 2% sodium hydroxide solution. Afterwards, the geometry was sanded to obtain a smooth surface. The manufactured geometry was then placed in a box, which was subsequently filled with transparent silicone (ELASTOSIL® RT 601 A/B, Wacker Chemie AG, Munich, Germany). At the top end of the IVC, a 1/8” PVC tubing (T) was placed perpendicular to the flow direction to function as a manometer. The geometry was connected to two different flow loops (Fig. 4): The first loop consisted of a flow sensor (Q) (MAG 6000, Danfoss A/S, Nordborg, Denmark) and a pump (P) (Deltastream DP2, XENIOS AG, Heilbronn, Germany), and was used to set the desired flow and pressure in the IVC and CIVs. The second loop consisted of a color-sensor box (CSB), a pump (P) (Deltastream DP2, XENIOS AG, Heilbronn, Germany) a flow sensor (Q) (SonoTT™ Clamp-On Transducer 3/8”, em-tec GmbH, Finning, Germany), and another pump (P) (Deltastream DP2, XENIOS AG, Heilbronn, Germany). Between the first pump and the flow sensor, two three-way valves (V) were placed. The valves could be either connected to each other, or, *via* a flow sensor (Q) (SonoTT™ Clamp-On Transducer 3/8”, em-tec GmbH, Finning, Germany), to a drain (first valve) or a dye reservoir (R) (second valve). Prior to the experiments, we ensured an equal distribution of the flow between both CIVs by measuring each branch individually.

The color-sensor box was manufactured in-house and consisted of an LED opposite of a color sensor (TAOS TCS230, AG, Premstaetten, Austria) in a light-blocking housing, which could be clipped on to a tube. The color sensor had four different channels, red green, blue, and clear, which were all used for the analysis. A fluid mixed from Voluven 6 and Voluven 10% (Fresenius SE & Co. KGaA, Bad Homburg, Germany) in a ratio of 2:5, resulting in a density of 1.031 kg/m^3^ and a dynamic viscosity of 3.62 mPas, closely resembling the parameters of blood used for the CFD simulations. A part of the fluid was dyed, using simplicol Fabric Dye expert Midnight Black (Brauns-Heitmann GmbH & Co. KG, Warburg, Germany), with a ratio of 11.5 g dye per 3 L of fluid.

Immediately before the experiments, we determined a calibration curve for the color sensor, by measuring different mixtures of clear and dyed fluid. The mixtures were 100, 50, 30, 25, 20, 12.5, and 0% of dyed fluid. Additionally, the sensitivity of the color sensor was assessed by incrementally adding dye to the clear fluid and recording the resulting signal. The first ratio that produced a sensor signal was defined as the minimum recirculation measurable. At the start of the experiments the loop was filled with clear fluid and the three-way valves were connected to each other. With this configuration, the flows and pressures were adjusted to match the values used in the CFD simulation: 2.0 L/min IVC flow, which results in 1.0 L/min for each CIV, 1.5 or 1.0 L/min graft flow, depending on the experiment, and a pressure of 6 mmHg at the top of the IVC. Subsequently, both three-way valves were switched simultaneously, whereby the dyed fluid was added to the loop, flowed through the return graft, and mixed with the clear fluid from the CIVs. The resulting fluid was measured with the color sensor and discarded. Every experiment was repeated five times.

## Results

### Anatomical Model

**Figure 5:**
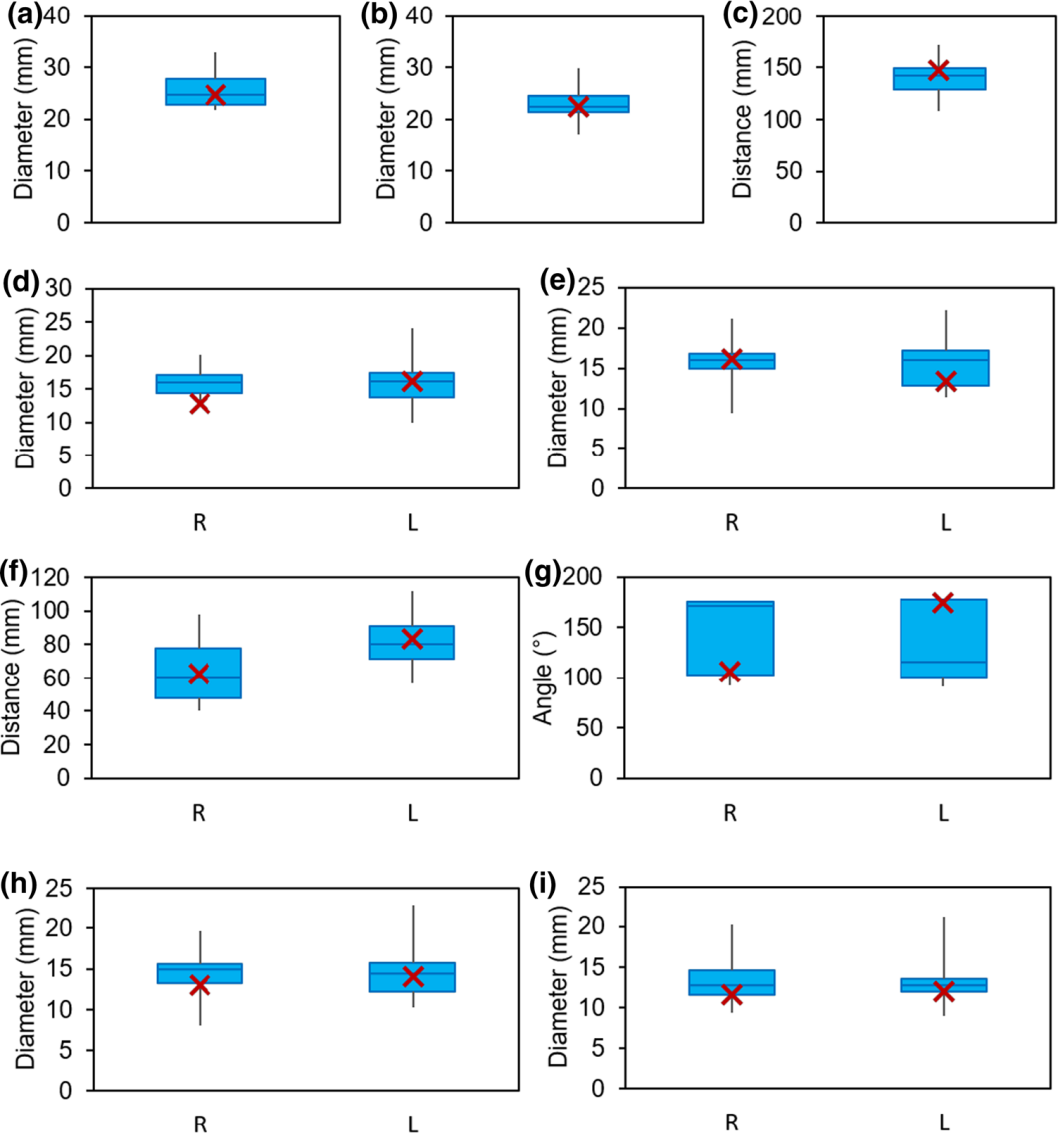
Boxplots of the anatomical measurements, wherein the value of the selected anatomy are marked with a red “X”: (a) Diameter D1, (b) Diameter D2, (c) Distance D1-D2, (d) Diameter D3 & D4, (e) Diameter D5 & D6, (f) Distances D3-D5 & D4-D6, (g) Angle G-L & G-R (h) Diameter D7 & D8, (i) Diameter D9 & D10.

The results of the anatomical analysis of the 18 image data sets are presented in Fig. 5. The mean diameter ± standard deviation of the IVC at the level of the first lumbar vertebra is 25.8 ± 3.3 mm and decreases on a mean length of 138.4 ± 17.8 mm towards the CIVs to a mean diameter of 22.9 ± 2.7 mm. Just before the convergence of the CIVs to the IVC, the right and the left CIV have a mean diameter of 15.9 mm with a standard deviation of 2.8 mm and 3.3 mm, respectively. The right CIV is on average 63.6 ± 18.0 mm long, whereas the left CIV is longer, with an average length of 81.4 ± 14.8 mm. The right and left vena iliaca externa show similar diameters just before the convergence into each CIV, 14.4 ± 2.4 mm and 14.3 ± 2.9 mm, respectively. The same applies for the diameters at the beginning of both right and left vena iliaca externa, as they show a diameter of 13.3 ± 2.6 mm and 13.1 ± 2.4 mm, respectively. The angles between the IVC and each CIV show the highest variances: The angle between IVC and right and left CIV are 142.0 ± 37.1° and 135.7 ± 37.4°, respectively. The angle between both CIVs shows a lower variance, with 83.3 ± 6.1°.

The performed analysis entailed two candidate anatomies for the subsequent CFD simulations: subject 3 and 10. Because of the limited field of view of subject 10, a measurement of D1 was not possible and therefore, we selected the anatomy of subject 3. The respective measurements of subject 3 are marked in Fig. 5.

## Computational Fluid Dynamics

**Figure 6:**
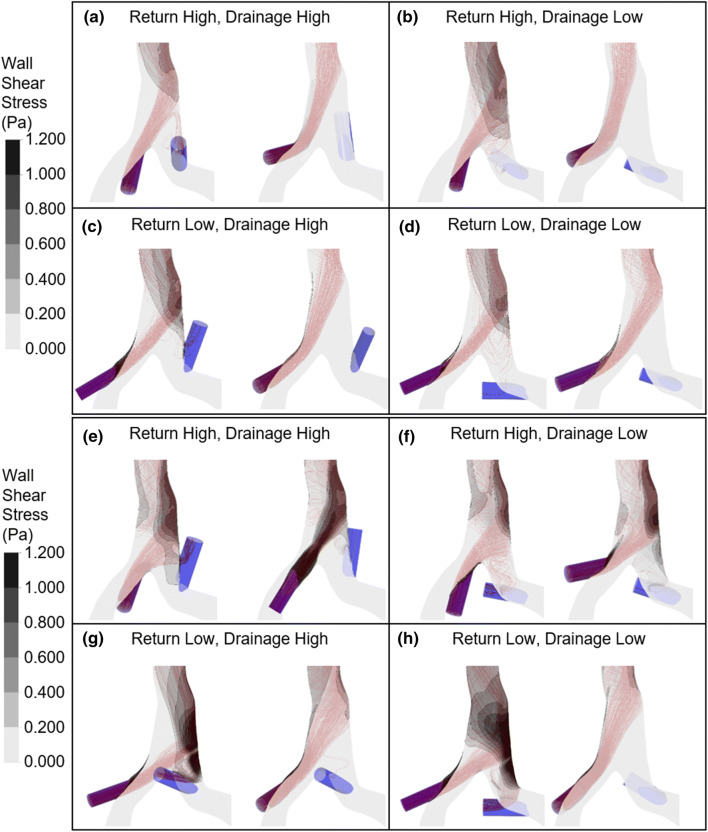
Streamlines and WSS contour plots of the highest (right) and lowest (left) recirculation for each graft configuration: (a)–(d) 100% graft flow, (e)–(h) 150% graft flow.

Figure 6 depicts the streamlines of the highest and lowest recirculation and contour plots of the WSS for each graft configuration. The graft flows of the worst-case scenario of 150% of the CIV flows leads to recirculations of up to 32.3%. In comparison, the graft flow of the critical scenario entails overall significantly lower recirculations with a maximum of 8.9%. The WSS contour plots show two areas prone to slightly elevated WSS on the vessel wall: one around the return graft and another one where an inflow jet hits the vessel wall. These areas of impingement seem to be more pronounced in the graft configurations with the maximum recirculation. Also apparent is an increase of the maximum WSS for the 150% graft flow compared to the 100% graft flow.

**Figure 7:**
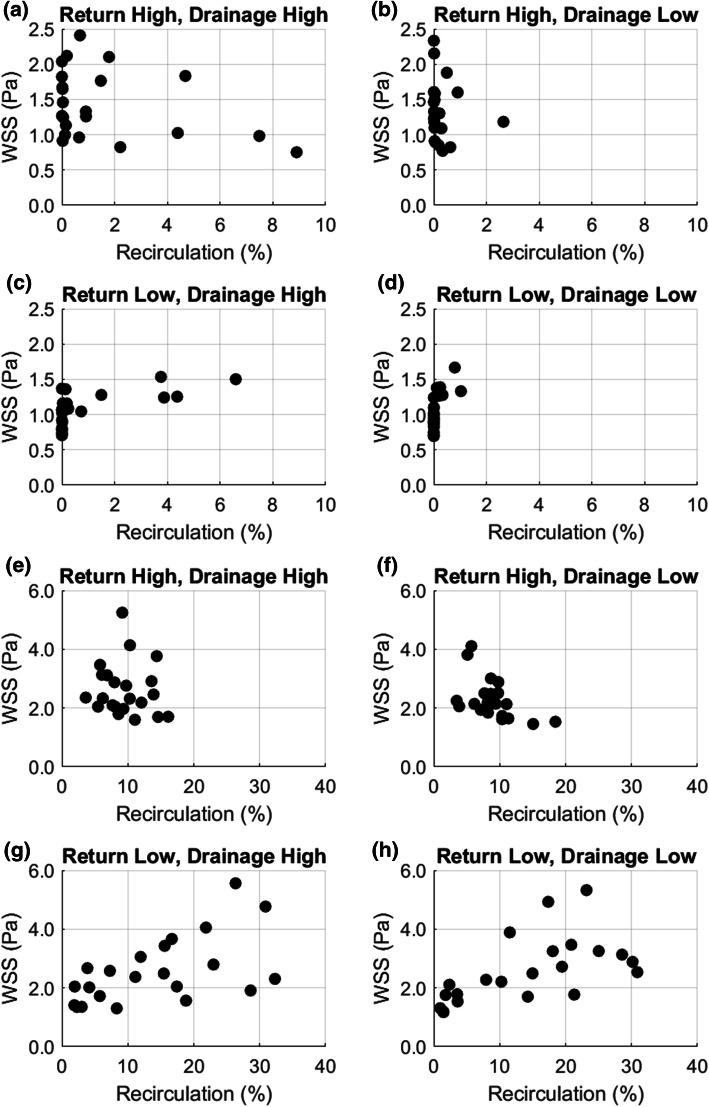
Scatter plots of maximum WSS dependent on recirculation: (a)–(d) 100% graft flow, (e)–(h) 150% graft flow.

The results of the sensitivity study for a graft flow of 150% identify the rotational angle of the return graft as the parameter that affects the recirculation most, with a mean value of 0.50, followed by the diameter of the return graft with 0.21. For a graft flow of 100%, the recirculation is most sensitive to the diameter of the return graft, with a value of 0.51, followed by the rotational angle of the inlet graft with 0.43. Additionally, a weak correlation between an increased recirculation and an increased WSS could be derived from Fig. 7. Also apparent in this figure, for a 150% graft flow, is a connection between the return graft in the lower location and an increased recirculation: The lower location of the return graft results in recirculations of more than 30%, whereas the higher location of the return graft entails maximum recirculations of mostly less than 20%. The location of the drainage graft, on the other hand, does not seem to have an influence on the recirculation. Contrary to this, for a 100% graft flow, the drainage graft location affects the amount of recirculation: The drainage graft in the higher location results in recirculations of more than 6%, whereas the drainage graft in the lower position entails recirculations of less than 3%. Here, the location of the return graft does not appear to impact the recirculation. The maximum WSS for the simulations with 150 and 100% graft flow did not exceed 5.57 Pa and 2.41 Pa, respectively. Hence, the decrease of the graft flow by 33%, decreases the maximum WSS by approximately 60%. Scatter plots of the recirculation dependent on the varied parameters (angle of drainage and return graft, as well as diameter of drainage and return graft) are provided in the appendix (Figs. 10–13).

## Experimental Validation

**Figure 8:**
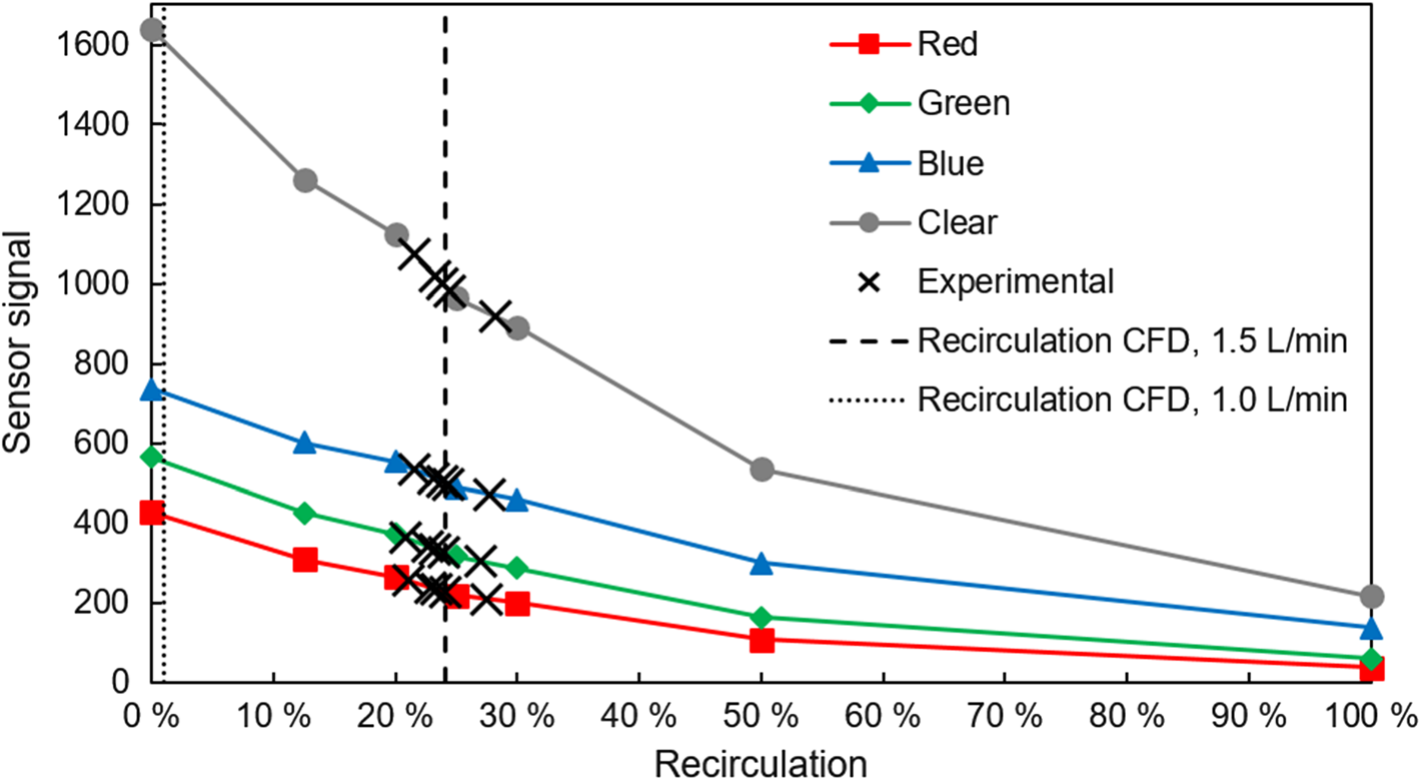
Calibration curves of the color sensor for the four channels with expected recirculation values calculated from CFD simulations (dashed vertical lines) and the results of the experimental validation (crosses).

**Figure 9:**
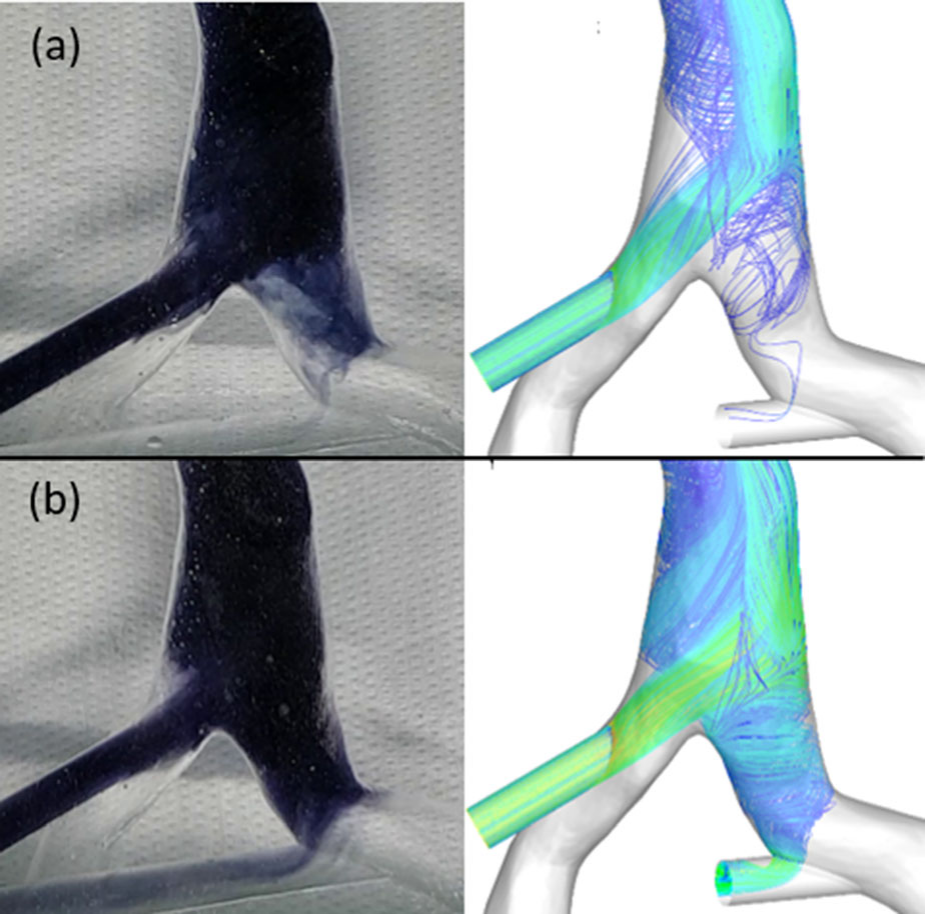
Qualitative comparison of CFD simulations and validation experiments for the configuration Rh_Dl and a graft flow of 1.0 L/min (a) and 1.5 L/min (b).

**Figure 10:**
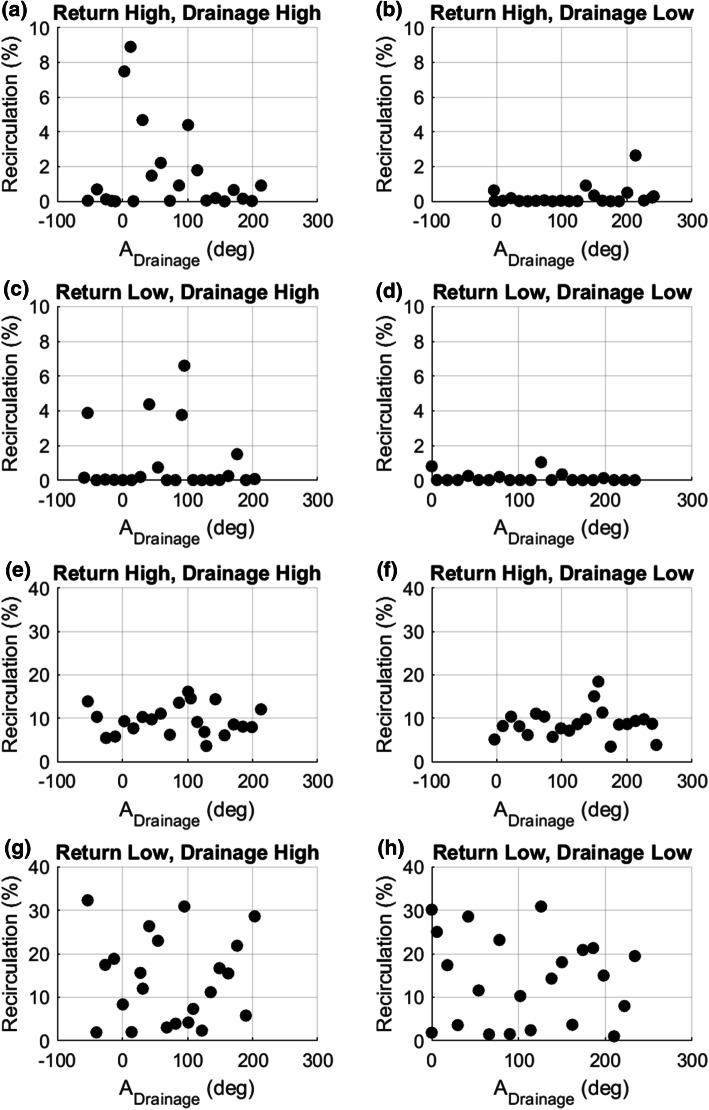
Scatter plots of recirculation dependent on the angle of the drainage graft: (a)–(d) 100% graft flow, (e)–(h) 150% graft flow.

**Figure 11:**
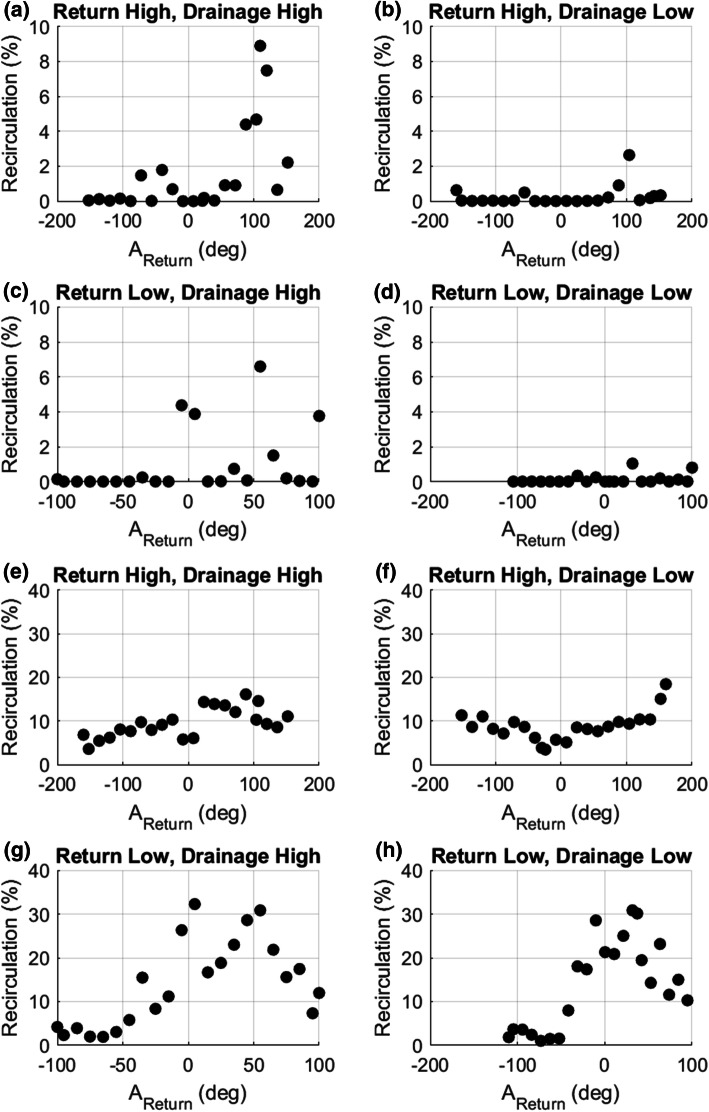
Scatter plots of recirculation dependent on the angle of the return graft: (a)–(d) 100% graft flow, (e)–(h) 150% graft flow.

**Figure 12:**
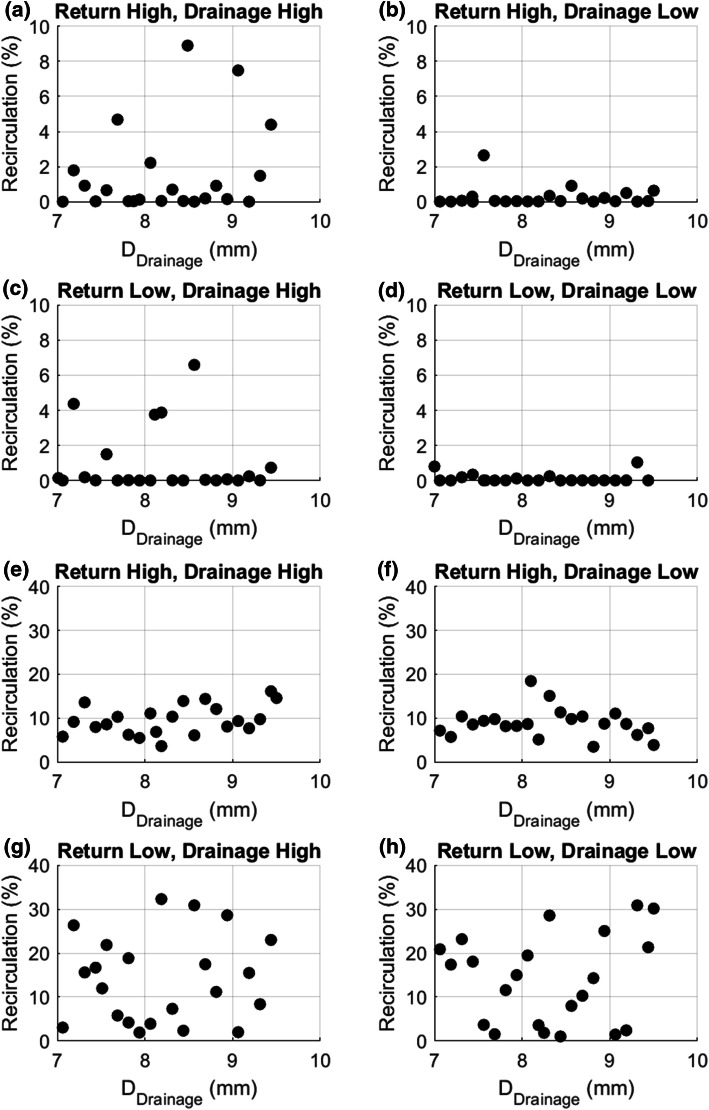
Scatter plots of recirculation dependent on the diameter of the drainage graft: (a)–(d) 100% graft flow, (e)–(h) 150% graft flow.

**Figure 13:**
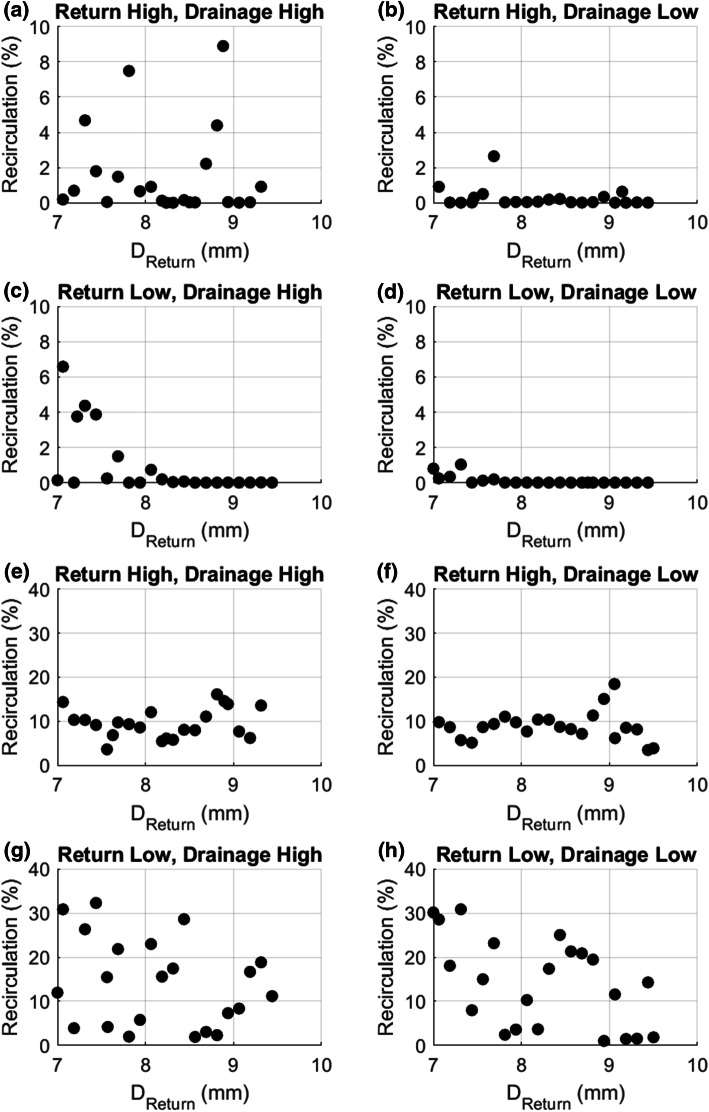
Scatter plots of recirculation dependent on the diameter of the return graft: (a)–(d) 100% graft flow, (e)–(h) 150% graft flow.

The calculated values for the recirculation from the CFD simulations are shown in Fig. 8: 24.04 and 1.0% for a graft flow of 1.5 and 1.0 L/min, respectively. The figure also depicts the calibration curves of the four color sensor channels. On the curves, the measured sensor signals in the five experiments for a graft flow of 1.5 L/min are matched with the corresponding recirculation. The measured values were 232.4 ± 16.8 (red), 334.3 ± 21.3 (green), 503.3 ± 22.6 (blue), and 998.3 ± 57.5 (clear). Converted into percentage recirculation using the calibration curve, these values correspond to 23.5 ± 2.4% (red), 23.3 ± 2.2% (green), 23.8 ± 2.3% (blue), and 23.9 ± 2.4%. The recirculation of a graft flow of 1.0 L/min did not yield any measurement values, because the sensitivity of the color sensor only allowed measurements of a minimum of 1.5% recirculation. A qualitative comparison of the CFD simulations with the experimental validation is shown in Fig. 9.

## Discussion

In the present study, we investigated the possibility of connecting an ECC for ECCO_2_R to the common iliac veins using grafts. First, the anatomy of the iliac veins was analyzed and an anatomical model for the subsequent CFD simulations was chosen. Second, the diameter of the grafts, the rotational angle around the native flow direction, and the height of the grafts were varied in the CFD simulations using the developed anatomical model, before an optimization regarding recirculation was performed. Third, we validated the CFD simulation experimentally in a silicone model of the iliac and cava veins by conducting dye tests.

The results of the anatomical measurements show relatively low variations, except for the angles between the CIVs and the IVC. Comparing the diameters along the progression of the IVC into the CIVs, and farther on the external iliac veins, entails decreasing values. Therefore, we consider the anatomical study as reasonable. However, our results are at the upper end of the values reported in the literature for CIV diameters: e.g. 11.2 ± 3.3 mm,^11^ 11.5 mm (range 8.7–13.9 mm),^39^ and 11.5 mm (range 6.3–16.1 mm).^37^ Additionally, our selected anatomy has a relatively small diameter of the right CIV and the angles between IVC and CIVs are only just inside the interquartile range. Nevertheless, this anatomy showed the lowest overall variance, which may allow a more general interpretation of the effects of the parameters, regardless of the patient-specific geometry.

The experimental validation for a 150% graft flow is in good agreement with the CFD simulations, with a mean deviation across all measurements and channels of 1.64%. This is corroborated by the experiments for the 100% graft flow: No recirculation was measurable, because the expected recirculation of 1.0% is less than the sensitivity of the color sensor, which is 1.5%. In addition, the qualitative comparison of the flow patterns (see Fig. 9), shows a large similarity. However, for the 100% graft flow experiments, there is a noticeable difference above the return graft: The results of the CFD simulations show an area on the left side of the IVC without any fluid from the return graft. On the contrary, the image of the experiments shows this area filled with dye. Therefore, fluid from the return graft must have entered this area. We assume that this happens due to transient effects induced by the switching of the two three-way valves at the start of the dye measurements.

Traditionally, particle image velocimetry (PIV) is used as a validation experiment for CFD simulations. With PIV the flow field can be visualized and the flow velocity can be measured. From this, the WSS can be determined, which is then compared with the simulations. However, PIV does not allow a distinction of different fluids and therefore a quantification of the recirculation is not feasible. Thus, we chose the described dye experiments to validate our simulations, because the recirculation was the primary focus of the study. However, the data from these experiments cannot be used to validate the simulated WSS results. For further experiments including a more detailed investigation of the WSS, PIV measurements should be chosen as additional validation experiments.

The reason for the predominant influence of the rotational angle of the return graft on the recirculation for a 150% graft flow is most likely related to “jet splitting”: If the inflow jet is directed at the opposing wall of the IVC, the impact on the wall splits the jet. One part flows upwards, in the native flow direction of the IVC. The other part of the jet is directed towards the left CIV and the drainage graft. Thereby, the reinfused fluid can reach the drainage graft and is subsequently transported back through the ECC. The degree of recirculation is then dependent on the magnitude of the impingement of the jet on the vessel wall. This assumption is supported by the correlation of the recirculation and the average WSS, because a stronger impingement of the jet on the wall entails a higher WSS in the impingement region.

Considering these aspects, we deduce that the strong influence of the rotational angle on the recirculation is directly connected to the strong influence of the impingement angle on the recirculation, because the impingement angle itself depends on the rotational angle of the return graft. Differently rotated grafts direct the inflow jet either more towards the wall of the IVC or in the native flow direction of the IVC. This can also explain the increased recirculation of the configurations with the return graft in the lower location: There are supposedly more positions in the lower location that are prone to the inflow jet hitting the vessel wall. The higher locations are less prone to the inflow jet hitting the vessel wall, because the angle of the CIV to the IVC affects this position less.

The sensitivity of the recirculation to the return graft diameter is likely due to decreased velocities of the inflow jet with larger diameters: The momentum of the inflow jet is decreased, which is why it can be more easily deflected by the native blood flow towards the IVC.

For the 100% graft flow simulation, the order of the sensitivities is reverse, with the rotational angle showing less influence on the recirculation. This can also be explained by the momentum of the inflow jet. The momentum is decreased even further, again allowing a better deflection of the inflow jet by the vessel flow. This is again supported by the WSS results, as they are lower as well.

Ultimately, for a 100% graft flow, there are more rotational angles of the return graft, in which the jet split is reduced or even avoided completely, than for a 150% graft flow. Consequently, the impact of the diameter of the return graft on the flow velocities becomes more notable. Also notable is the increased recirculation with a higher location of the drainage graft, which we also attribute to the decreased flow velocities. Hence, the resulting decreased momentum of the inflow jet results in a weaker jet split, causing a lower momentum of the jet directed towards the drainage graft. Therefore, the drainage graft in the higher location, closer to the impingement region, is reached more easily by the flow from the return graft before it can be redirected by the native flow from the CIV.

The comparison of the WSS contour plots of the highest and lowest recirculation in Fig. 6 supports our hypothesis described above, as the graft configurations with maximum recirculation are associated with more pronounced areas of impingement. Nonetheless, Fig. 7 indicates merely a weak correlation between WSS and recirculation. This could be attributed to the conflicting influence of the diameter of the return graft. Since one area of high WSS is located around the return graft, the maximum WSS in this area would increase with smaller graft diameters, as this induces higher velocities compared to grafts with larger diameters. Therefore, combinations of a small graft diameter and an angle of the return graft in a position that entails a low recirculation could account for graft configurations that weaken the correlation between WSS and recirculation.

There are several limitations of this study. (1) Even though we selected a reference model with the lowest deviation from the mean, our study was performed only for this specific anatomy. Therefore, generalizations of our conclusions should be treated conservatively. (2) Our study includes several simplifications regarding the graft connections, as they are implemented in simplified form as ideally straight tubes with a constant angle of 25°. More realistic graft designs, for example tapered, curved, or hooded grafts, as well as changing angles due to the movement of the patient, were not considered. These aspects can critically impact the flow behavior and thus, the recirculation and the WSS, which limits the generalization of the results. Additionally, our study was performed in a model with rigid walls and without dynamic changes in flow or pressure. For a long-term application with ambulated patients, these aspects should be considered in subsequent studies, because events like the collapsing of a graft might exclude the proposed connection from a clinical application. (3) The study is further limited by modeling the blood flow as a Newtonian fluid. In reality, blood is a non-Newtonian fluid and shows shear-thinning behavior with increasing shear rates.^5^ However, we chose the Newtonian model to allow a reasonable comparison to the validation experiments as there are no standard non-Newtonian blood-analogue fluids. Furthermore, the appropriate application of the various non-Newtonian models is subject to discussion in the literature^10^,^40^ and several studies confirm that Newtonian rheology provides a reasonable prediction for blood flow behavior.^10^,^26^,^28^,^31^,^35^,^41^ (4) Only the grafts themselves or the veins limited the positioning of the grafts; no other anatomies were taken into account, especially not of the traversing right common iliac artery. Therefore, it is possible that the resulting ideal graft configurations cannot be surgically applied in a clinical scenario. (5) A compromise between mesh size and computing time had to be found. As quantitative WSS evaluations require extremely fine meshes towards the wall, we decided to limit the evaluation of WSS values in this study to a qualitative comparison of the configurations. For quantitative assessment of the WSS, a refined wall mesh as well as the consideration of WSS in the mesh sensitivity study are necessary.

In conclusion, our study shows that connecting a VV ECCO_2_R to the CIVs using grafts may be possible without recirculation. Especially for a graft flow of 100% CIV flow the majority of graft configurations entail a negligible recirculation. Thus, optimum positioning of the grafts can completely avoid recirculation: The return graft should be aligned with the native flow direction of the IVC so that the inflow jet does not hit a vessel wall. Additionally, a larger graft diameter is preferable, both because of lower WSS and because of a lower risk of recirculation. Lastly, a location of the drainage graft farther away from the IVC is favorable. The angle and diameter of the drainage graft show a negligible influence on the recirculation. Especially for even smaller ratios of graft and CIV flows, as used for ECCO_2_R,^25^ we assume that recirculation becomes even less likely.

The results show that this new connection has a potential for further investigation. The next step should be a more detailed analysis of few anatomically feasible graft configurations, including pulsatile flow and flexible vessel walls. Additionally, experiments with flexible silicone tubes, pulsatile flow, and realistic designs of the grafts could be performed. Ultimately, the feasibility of the new connection will have to be investigated in *in*-*vivo* experiments.

## Appendix

See Figs. 10, 11, 12, and 13.
